# Supplementary material for: Drug repurposing for aging research using model organisms
Source: Aging Cell. 2017 Jun 16;16(5):1006–15. doi: 10.1111/acel.12626 (PMC5595691; doi:10.1111/acel.12626)
Supplement: Supplementary file 7 — Data S1 Zip‐Archive of all report cards. [file ACEL-16-1006-s007.zip › RC_JZG.pdf]

JZG

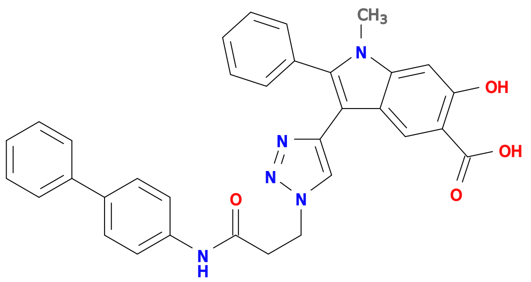

Database identifiers
ChEMBLCompound CHEMBL590043

Ranking

	Rank	Score
Drosophila	659/697	0.101
C. elegans	547/591	0.031

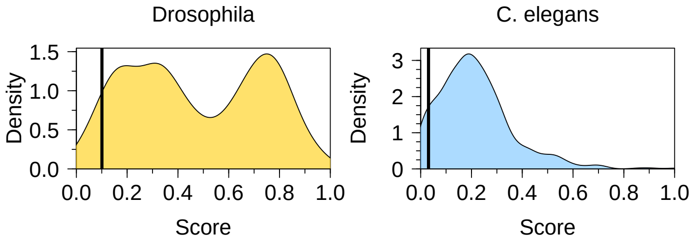

	Ageing implication	Domain conservation	Binding site conservation	Binding affinity	Bioavailability	Lipinski	Promiscuity	Purchasability	Drug approval	**Total**
Drosophila	0.81	0.425	1.0	0.65	(0.9)	-0.1	-0.0	0.0	0.0	0.101
C. elegans	0.81	0.641	1.0	0.65	0.389	-0.1	-0.0	0.0	0.0	0.031

Names

No synonyms found

Roles

ChEBI entry None has no roles

Status

Approved drug (according to ChEMBL)	No
Number of Rule of 5 violations	2
Binding affinity to original target in log units (RF-Score prediction)	5.62
Burns *C. elegans* bioavailability prediction	1.36

Compound Target Characteristics

Tyrosine-protein phosphatase non-receptor type 11

Best gene implication in ageing for this target family came from gene G5EC24 via mapping the annotation from WormBase WBGene00004214 annotated in WormBase 2014-03-11. Annotation

GO 8340 (determination of adult lifespan) was Inferred from Mutant Phenotype

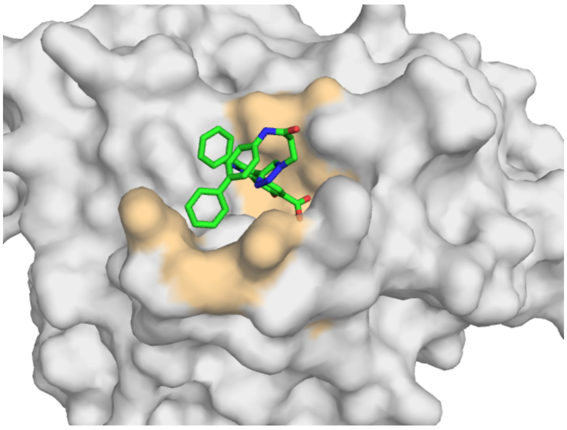

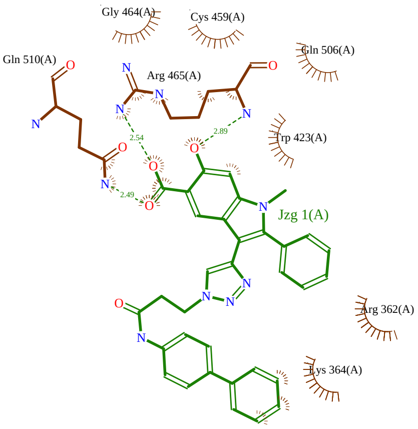

protein	amino acids contacts (binding site)
PDB:3o5x:chainA:Q06124	R K W C G R Q Q
sp:Q06124:PTN11_HUMAN	R K W C G R Q Q
sp:P41499:PTN11_RAT	R K W C G R Q Q
sp:P35235:PTN11_MOUSE	R K W C G R Q Q
sp:P29349:CSW_DROME	R K W C G R Q Q
tr:G5EC24:G5EC24_CAEEL	R K W C G R Q Q

	whole protein		domain-based		contact-based	
protein	ident	simil	ident	simil	ident	simil
PDB:3o5x:chainA:Q06124	1.0	1.0	1.0	1.0	1.0	1.0
sp:Q06124:PTN11_HUMAN	1.0	1.0	1.0	1.0	1.0	1.0
sp:P41499:PTN11_RAT	0.99	1.0	0.99	1.0	1.0	1.0
sp:P35235:PTN11_MOUSE	0.99	1.0	1.0	1.0	1.0	1.0
sp:P29349:CSW_DROME	0.41	0.57	0.39	0.57	1.0	1.0
tr:G5EC24:G5EC24_CAEEL	0.32	0.64	0.37	0.66	1.0	1.0

csw (FBgn0000382) associated phenotypes

dominant, germline clone, heat sensitive, increased cell death, lethal - all die before end of pupal stage, maternal effect, rescuable maternal effect, somatic clone, some die during pupal stage

(Information from FlyBase)

csw (UniProt:P29349) annotation

Function: Required in all receptor tyrosine kinase signaling pathways. Functions downstream of the receptor tyrosine kinase torso, acting in concert with D-Raf via tailless. Also functions downstream of Egfr (epidermal growth factor receptor) and btl (fibroblast growth factor receptor). The SH2 domain suggests that csw effects its role by mediating heteromeric protein interactions. Maternally required for normal determination of cell fates at the termini of the embryo. Required for cell fate specification of the ventral ectoderm, in the developing embryonic CNS and for embryonic tracheal cell migration. Functions during imaginal development for proper formation of adult structures such as eyes, aristae, L5 wing vein and the tarsal claw. (PubMed:1638629, PubMed:8948575).
Subcellular location: Cytoplasm.
Tissue specificity: Expressed uniformly throughout all tissues during embryogenesis. (PubMed:1638629).
Developmental stage: Expressed throughout development. (PubMed:1638629).
Miscellaneous: The PTPase domain is interrupted by a PTPase insert which shares no homologies with other PTPase proteins. This PTPase insert is reminiscent of the kinase insert within the kinase catalytic domains of several receptor tyrosine kinases.

(Information from UniProt)

ptp-2 (UniProt:G5EC24) annotation

Subcellular location: Cytoplasm ECO:0000256—PIRNR:PIRNR000929.

(Information from UniProt)
